# Magnitude and sources of bias in the detection of mixed strain *M. tuberculosis* infection

**DOI:** 10.1016/j.jtbi.2014.12.009

**Published:** 2014-12-29

**Authors:** Giacomo Plazzotta, Ted Cohen, Caroline Colijn

**Affiliations:** a Imperial College London, United Kingdom; b Brigham and Women's Hospital, Harvard School of Public Health, United States

**Keywords:** Study designs aimed to detect mixed infection, Majority and minority strain, Prevalence of mixed infection, Posterior distribution of the prevalence of mixed infection, Estimates of the prevalence of mixed infection

## Abstract

High resolution tests for genetic variation reveal that individuals may simultaneously host more than one distinct strain of *Mycobacterium tuberculosis*. Previous studies find that this phenomenon, which we will refer to as “mixed infection”, may affect the outcomes of treatment for infected individuals and may influence the impact of population-level interventions against tuberculosis. In areas where the incidence of TB is high, mixed infections have been found in nearly 20% of patients; these studies may underestimate the actual prevalence of mixed infection given that tests may not be sufficiently sensitive for detecting minority strains. Specific reasons for failing to detect mixed infections would include low initial numbers of minority strain cells in sputum, stochastic growth in culture and the physical division of initial samples into parts (typically only one of which is genotyped). In this paper, we develop a mathematical framework that models the study designs aimed to detect mixed infections. Using both a deterministic and a stochastic approach, we obtain posterior estimates of the prevalence of mixed infection. We find that the posterior estimate of the prevalence of mixed infection may be substantially higher than the fraction of cases in which it is detected. We characterize this bias in terms of the sensitivity of the genotyping method and the relative growth rates and initial population sizes of the different strains collected in sputum.

## 1. Introduction

Tools for the genetic analysis of *Mycobacterium tuberculosis*, the causative agent of human tuberculosis (TB), have fundamentally altered our understanding of the natural history of this pathogen. The ability to distinguish isolates has shown that individuals can be re-infected with *M. tuberculosis*, and this poses clear challenges for vaccine development since even natural infection at best provides partial immunity. Furthermore, the advent of high resolution tests for genetic variation has revealed that individuals may *simultaneously* harbor infections with more than one distinct strain of *M. tuberculosis* (Warren et al., 1999; Sola et al., 2003; Kremer et al., 1999; van Embden et al., 1993; Imaeda, 1985). This phenomenon, which we will refer to as “mixed infection”, has been linked with poor treatment outcome when the co-infecting strains differ with respect to drug susceptibility (van Rie et al., 2005; Hingley-Wilson et al., 2013) and is predicted to influence the impact of population-level interventions against tuberculosis (Cohen et al., 2008; Rodrigues et al., 2007; Colijn et al., 2009; Sergeev et al., 2011; Mills et al., 2013).

Accurate estimates of the frequency with which mixed infections occur are therefore critical to understand how mixed infections impact both the natural history and the dynamics and control of this infection. However, the detection of mixed infections is challenging (Hingley-Wilson et al., 2013), even with tools that have high sensitivity for detecting minority strains and adequate resolution to discriminate between closely related (but genetically distinct) pathogens. As discussed in detail in a recent review (Cohen et al., 2012), there are many opportunities to fail to detect a mixed infection that is actually present in a host, because a minority strain might not be harvested in the collected clinical specimen, might be lost during the process of specimen transport and handling, and might fail to be detected by the particular genotyping method employed.

Despite these clear opportunities to miss the detection of true mixed infections, among the several dozen studies available, it has been found that mixed infections were often detected in as many as 10–20% (Cohen et al., 2012; Hanekom et al., 2013; Huang et al., 2010; Navarro et al., 2011) of cases in areas where the incidence of TB is high (Cohen et al., 2012). Since we believe that this statistic may underestimate the prevalence of mixed infections, we have developed a mathematical model to understand the potential sources of bias in estimates of the prevalence of mixed infection and to provide bounds for reasonable uncertainty as to the actual prevalence of mixed infections given the observed prevalence and knowledge of the laboratory protocol employed to detect mixed infections.

## 2. Methods

**Fig. 1.**
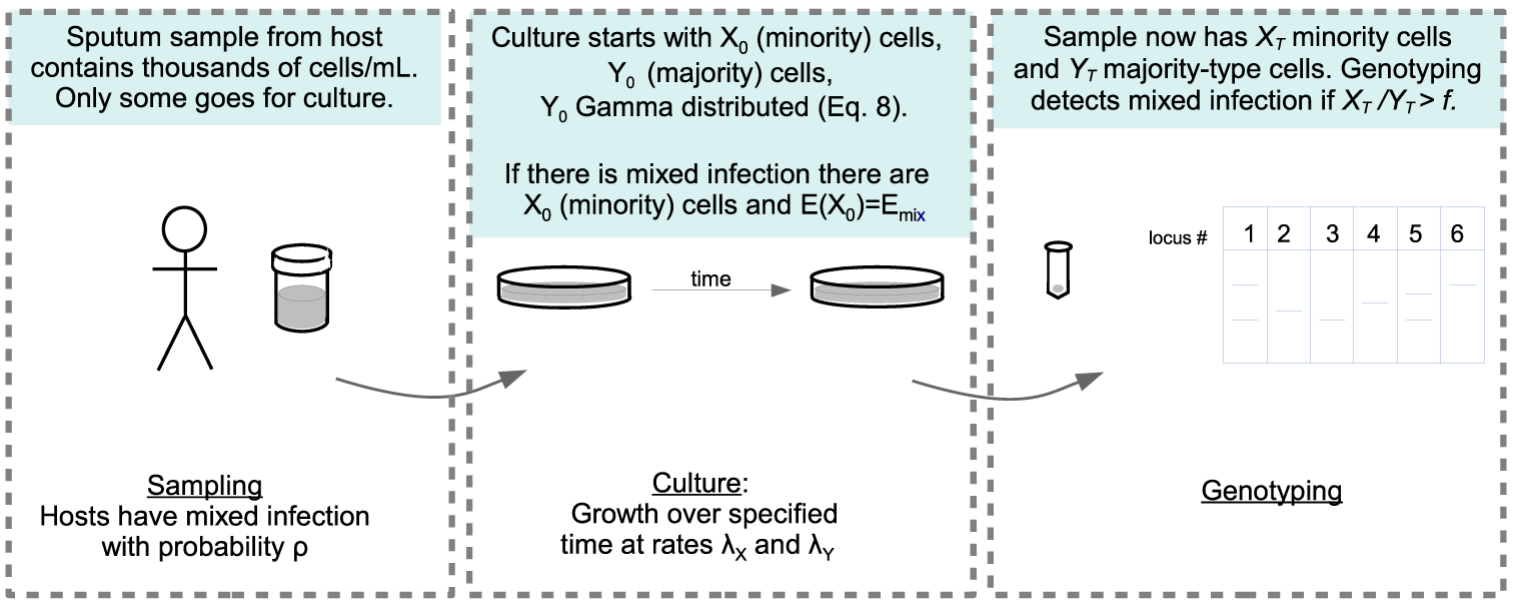
Schematic of the process of sampling, culture and genotyping.

Although the designs of previous studies for detecting mixed strains have differed in important ways (Cohen et al., 2012), for the purposes of this analysis, we have generalized the study design to include several steps common to nearly all of these investigations:

1. Specimen collection from the patient (samples of 0.25 mL).
2. Specimen growth in culture.
3. Sampling of bacterial isolates from culture and extraction of mycobacterial DNA.
4. Analysis of mycobacterial DNA (see Fig. 1).

Here we focus our analysis on bias that might arise in the detection of mixed infections related to steps 2–4 above. That is, we do not consider the bias that might result from failing to collect a minority strain from an individual, and instead we focus here on the bias that arises from failing to detect a minority strain after it has actually been collected from a patient. This is not meant to indicate that we think failing to collect a minority strain from a patient does not contribute to the underestimation of the prevalence of mixed infection. Rather, this approach allows us to provide estimates on the bias that is associated with the laboratory procedures that are distal to specimen collection. We comment further on this issue in the discussion. The probability that mixed infection is detected can be decomposed as

(1) P(detect)=P(detect∣mixed infection present)P(mixed infection present).

We define the prevalence of mixed infection to be the fraction of individuals with TB disease that are simultaneously infected by more than one distinct strain. Here we define strains by their ability to be discriminated from each other by the particular genotyping test used. Our aim is to estimate the prevalence of mixed TB infection in a population, ρ≔P(mixed infection present), from a set of data consisting of measurements aiming to detect mixed infection in individuals. To do this, we characterize m≔P(detection∣mixed infection present) by modelling laboratory handling and subsequent growth of bacilli in culture. We use a stochastic model of specimen handling and growth where cell numbers are small, and a deterministic model otherwise. The model inputs are the distributions of the numbers of cells in the samples, and the growth rates of minority- and majority-type bacilli. We apply a Bayesian approach to find the posterior distribution of *ρ*, the prevalence of mixed infection, given data from genotyping analysis of mycobacterial DNA collected after division of sputum and subsequent solid culture.

*Specimen handling*: The mathematical framework developed in this section is based on three assumptions regarding the handling protocol and the specimen – i.e. the sputum sample – from an individual:

1. Each specimen contains at least one strain of *M. tuberculosis*, and may contain more (but we only model detection of two at most). The strain with more bacilli in the specimen is called the majority strain, and the other the minority strain.
2. Each specimen is handled similar to any other and in two phases: sub-division and growth. Sub-division consists of dividing the sputum sample into *d* groups (only one of which is then cultured). Growth refers to the culture of one of the portions of the sputum sample, over a fixed time *T*.
3. In both sub-division and growth, the majority and minority strains are assumed to behave independently.

We use *X* and *Y* to indicate the number of minority and majority strain cells, respectively; if *X* and *Y* are indexed, the index specifies the time. For example, *X*_0_ is the initial number of minority strain cells and *Y_T_* is the number of majority strain cells at time *T*, after sub-division and growth.

### 2.1. The minority strain

When the sample is collected, we assume that it contains *X*_0_ minority strain cells. During sub-division, to select a portion 1/*d* of the sample, each cell is chosen with probability 1/*d* or rejected with probability 1 – 1/*d*. Therefore the total number of bacteria after sub-division follows the binomial distribution Bin(*X*_0_, 1/*d*). Growth is modelled with a birth-only process with birth rate *λ_X_* over a time *T*. We choose a birth-only process because the death rate is believed to be negligible in comparison to the birth rate in culture, and because it is preferable to minimize the complexity of the model. Birth processes are characterized by a negative binomial distribution (Bailey, 1964, p. 87); in this case, as the process starts with Bin(*X*_0_, 1/*d*) cells from the sub-division phase, it follows that the distribution of *X_T_* is NegBin(Bin(*X*_0_, 1/*d*), 2^*−λ_X_T*^). Using the law of total probability, the explicit distribution for the number of minority cells after time *T* is found to be

(2) $$P(X_{T}=k|X_{0})=\sum_{i=1}^{\text{min}(X_{0},k)}\left(\begin{array}{c} k-1\\ i-1 \end{array}\right)\left(\begin{array}{c} X_{0}\\ i \end{array}\right){\left(\frac{1}{d}\right)}^{i}{\left(\frac{d-1}{d}\right)}^{X_{0}-i}p_{X}^{i}(1-p_{X}{)}^{k-i},$$

where *p*_*X*_ = 2 ^*−λ_X_T*^, with *λ*_*X*_ being the growth rate and *T* the growth time; we refer to the supplement for the derivation. Eq. (2) can be rather impractical because it presents computational challenges due to the size of the binomial coefficients. For this reason, we found an asymptotic approximation:

(3) $$P(X_{T}=k|X_{0})\approx C(1-p_{X}{)}^{k}(k-1{)}^{l(X_{0}-1)}\;\text{for}\;k\to \infty ,$$

where *l* and *C* are constant with respect to *k*. Interestingly *l* is also independent from *λ_X_*, *X*_0_, and *T*, hence it is specific to the handling protocol (see Supplement for details).

### 2.2. The majority strain

The majority strain is sub-divided and cultured along with the minority strain; following the same reasoning as in Section 2.1, the distribution of *Y_T_* is found to be

(4) $$P(Y_{T}=k|Y_{0})=\sum_{i=1}^{\text{min}(Y_{0},k)}\left(\begin{array}{c} k-1\\ i-1 \end{array}\right)\left(\begin{array}{c} Y_{0}\\ i \end{array}\right){\left(\frac{1}{d}\right)}^{i}{\left(\frac{d-1}{d}\right)}^{Y_{0}-i}p_{Y}^{i}(1-p_{Y}{)}^{k-i},$$

where *p_Y_* = 2^−*λ_Y_T*^ and *λ_Y_* is the growth rate of the majority strain. As in the previous section, Eq. (4) is impractical, but here because *Y*_0_ is assumed to be large (Core Curriculum for Disease Control; Palaci et al., 2007) – O(1000) – it is possible to use the Weak Law of Large Numbers to approximate the distribution (4) with a normal distribution (see the Supplement for details):

(5) $$P(Y_{T}=k|Y_{0})\approx N(k;\mu ,\sigma^{2}),$$

where

$$\mu =\frac{Y_{0}}{6}2^{\lambda_{Y}T}\;\;\text{and}\;\;\sigma^{2}=\frac{Y_{0}}{d}2^{\lambda_{Y}T}(2^{\lambda_{Y}T}-1)+\frac{Y_{0}d(d-1)}{d^{2}}4^{\lambda_{Y}T}.$$

We use Eqs. (3) and (5) to calculate the distributions of *X_T_* and *Y_T_* numerically in the next sections.

### 2.3. The conditional prevalence of mixed infection

After the phases of sub-division and growth in culture, genotyping is performed on DNA extracted from mycobacterial cells. In this paper we assume that the genotyping test performed is mycobacterial interspersed repetitive unit-variable number tandem repeat (MIRU-VNTR) typing (Supply et al., 2001). MIRU-VNTR typing is a convenient methodology to detect mixed infections, as multiple alleles at multiple loci are usually interpreted as the presence of mixed infection (Supply, 2005). Clearly, in order to detect a minority strain by MIRU-VNTR or any other method, the minority strain must be present in sufficient numbers. We define the threshold *f* as the minimum value of the proportion *X_T_*/*Y_T_* at which the minority strain, thus mixed infection, is detectable by MIRU-VNTR typing. It is convenient to introduce the new random variable *D* (for detection) which is defined by

(6) $$D=1\Leftrightarrow X_{T}∕Y_{T}>f\;\;\text{and}\;\;D=0\Leftrightarrow X_{T}∕Y_{T}<f.$$

where *D* is a Bernoulli random variable that is used to model the positive or the negative result of the test for mixed infection for each sputum sample.

Up to this point, we have analysed the dynamics of a single sample. To estimate the prevalence of mixed infection, we need to link our model to the outcome of a study aimed to detect mixed infection. Suppose there are *n* individual patients in the study and each of their sputum samples is sub-divided and cultured, and then tested for mixed infection. Let the outcome be denoted *D_j_*, for *j* = 1..*n*. The total number of detected mixed infection is $S_{D}≔\sum_{j=1}^{n}D_{j}$. Because the *D_j_*s are Bernoulli, it follows that

(7) $$P(S_{D}=k|X_{0},Y_{0})=\text{Binomial}(k;n,P(D=1|X_{0},Y_{0})),$$

where we recall that $P(D=1|X_{0},Y_{0})=P(X_{T}∕Y_{T}>f|X_{0},Y_{0})$.

### 2.4. Distributions of X_0_ and Y_0_

Eq. (7) is the distribution of the total number of detected mixed infections in *n* individuals, in a study that satisfies the initial assumptions outlined at the start of Section 2. To perform computations and statistical inference it is necessary to derive a distribution of *S_D_* that is not conditional on *X*_0_ and *Y*_0_. We have chosen particular distributions for these inputs, but the overall arguments we make about the effects of stochastic growth and low starting cell numbers are not specific to these particular choices.

The number of majority type cells *Y*_0_ is relatively large (Core Curriculum for Disease Control; Palaci et al., 2007) in the samples, with the order of magnitude 10^3^. Because *Y*_0_ is a discrete random variable we choose a discretized gamma distribution. The shape and scale parameters are also chosen to provide a reasonable expected value and variance: recall that the sputum sample is 0.25 mL and the concentration is 5000–10,000 per mL (Core Curriculum for Disease Control):

(8) $$P(Y_{0})=CDF_{Gamma(70,25)}(k)-CDF_{Gamma(70,25)}(k-1),$$

where *CDF* stands for the Cumulative Distribution Function.

The number of minority strain cells in the sample (*X*_0_) is likely to be variable. It will depend on many factors, including the dynamics of bacterial populations in the host, the time of reinfection and the distribution of cell types over different TB lesions. These factors may be elucidated in the future in studies using DEP frequency or single cell technologies, but at the moment there is very little information available to inform us as to the numbers of cells present in sputum samples from diverse infections.

When a minority strain is present, we do not have empirical information about the numbers of minority cells likely to be found in the sputum. We choose a class of distributions parametrized by their expectation *E_min_*, for the probability $P(X_{0}=k|X_{0}\geq 1)$ of finding *k* minority cells in the sample given that the host has two or more strains. The numbers of minority and majority cells in sputum will depend on a complex series of growth limitations imposed by the host during the course of infection, the relative timing of infection, the extent of in-host competition between the strains, the time that has elapsed before the patient comes to clinical attention and the non-random sampling of the in-host population in sputum. The inoculum for each strain of TB is likely to consist of a relatively small number of bacilli (Balasubramanian et al., 1994), and each strain presumably undergoes a period of exponential growth at some stage. So it is likely that a substantial difference in the robustness of the two strains in the host would lead to the less robust strain either being out-competed or being present in vanishingly small fractions in the host; a minority strain would either be “drowned out” in the exponential phase, or would suffer losses through the complex course of infection if it were not sufficiently robust. Such hosts would never be detected as mixed infections. For these reasons, to maintain high enough cell numbers to comprise ≈ 1% of a sputum sample, any minority strain will likely need to be a fairly strong in-host competitor. Conversely, when more than 2–5% of a sputum sample are minority strain bacilli, they are highly likely to be detected (and this will happen only for highly robust strains that achieve a very strong balance of cell numbers in the host). The problem of bias is most relevant when a minority strain is a robust enough competitor to rise to high enough levels that there is any change of detection, but not so high that detection is effectively certain. Accordingly, we investigate the range of *E_min_* in which minority strains comprise between 0 and 2% of the population of bacilli in the sputum i.e. *E_min_* ≤ 40.

Furthermore it must be taken into account that the prevalence of mixed infection corresponds to the probability $P(X_{0}\geq 1)$ of mixed infection present in the sample, that we called *ρ*. Note that *ρ* is fundamental for this study, as it is the parameter to be estimated. We use a Poisson distribution for *X*_0_, parametrized by *E_min_*:

(9) P(X0=k)={1−ρifk=0ρ⋅(Emin−1)k−1(k−1)!e−Emin+1ifk≥1}.

### 2.5. Posterior distribution of the prevalence of mixed infection

In this section we use the Bayesian inference to derive the distribution for the real prevalence of mixed infection, *ρ*, and we present an estimate of such prevalence. At first it is necessary to evaluate the probability P(D=1). The law of total probability can eliminate the condition on *X*_0_ and *Y*_0_ of $P(D=1|X_{0},Y_{0})$ in Eq. (7) using $P(X_{0})$ and $P(Y_{0})$ from Eqs. (8) and (9) respectively. Note that because the distribution of *X*_0_ is linear in *ρ*, the distribution of *X_T_* and the probability P(D=1) are also linear in *ρ*; this fact reflects the initial decomposition in Eq. (1). It follows that

(10) $$P(D=1)=P(X_{T}∕Y_{T}>f)=m\rho ,$$

where the slope *m* represents the probability P(detect∣mixed infection present) in (1); it depends on the parameters *λ_X_*, *λ_Y_*, *E_min_* and *T* and is calculated numerically using the law of total probability (we refer to the Supplement for further details). Because *D* has a Bernoulli distribution with probability *mρ*, the distribution of *S_D_* is binomial; therefore the probability of detecting *n_mix_* mixed infection in a study involving *n* patients is a binomial with *n_mix_* successes over *n* trials and with success probability *mρ*.

**Fig. 2.**
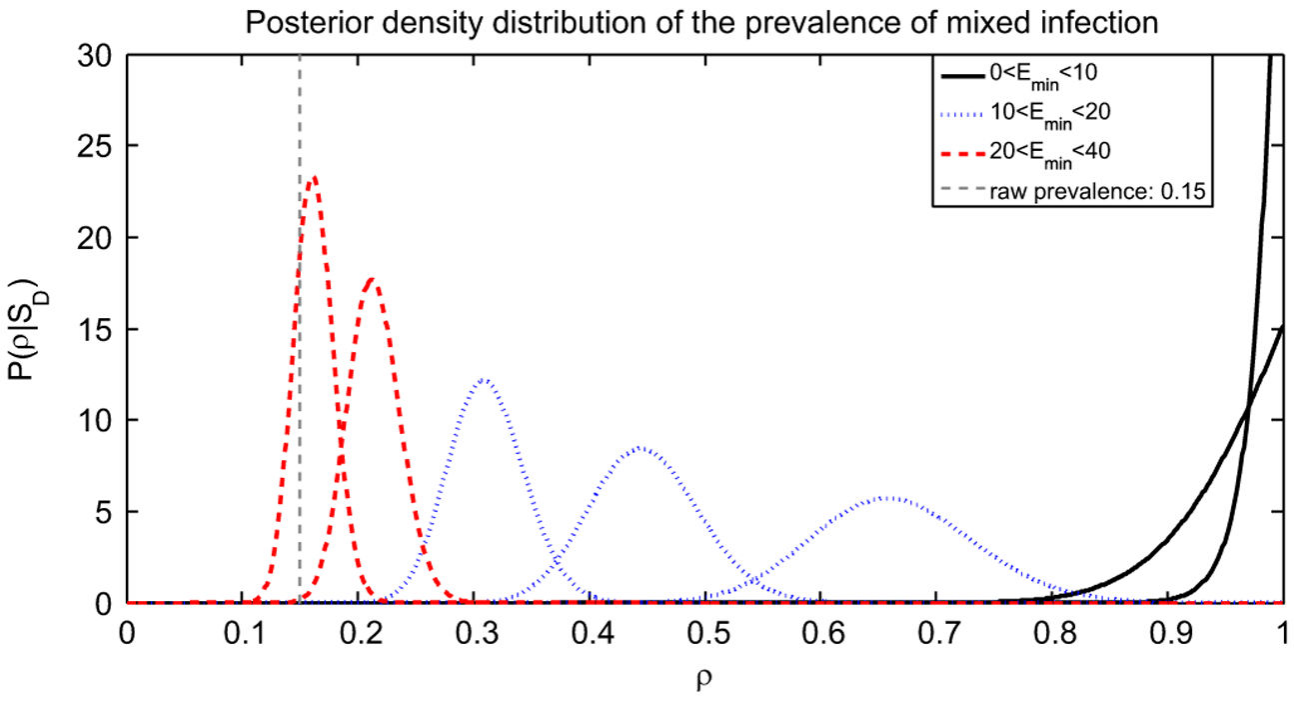
Posterior density distributions of the prevalence of mixed infection $P(\rho |S_{D})$ when both the growth rates for minority and majority type cells equal 1 and for different values of the expected number of minority cells in sputum *E_min_*. Bearing in mind that the larger the *E_min_* is, the smaller the mean of the distribution is, the values of *E_min_* that we used are 39, 25, 18, 14, 11, 8, 4. We considered *n* = 500 patients, *n_mix_* = 75 of whom are detected with mixed infection. A naive estimate from the data would indicate a mixed infection prevalence of approximately *n_mix_*/*n* = 15%, corresponding to *ρ* = 0.15. However the posterior distribution has mean close to 0.15 only if *E_min_* is large (*E_min_* > 40). The posterior distributions have a much higher mean as *E_min_* decreases.

In Bayesian notation, the binomial distribution of *S_D_* is the likelihood. We set an uninformative Beta prior distribution because it is a conjugate prior for the binomial (we refer to the Supplement for further details). This lead to the following posterior:

(11) $$P(\rho |S_{D})=\frac{(m\rho {)}^{n_{mix}}(1-m\rho {)}^{n-n_{mix}}}{B_{m}(n_{mix}+1,n-n_{mix}+1)},$$

where $B_{m}(n_{mix}+1,n-n_{mix}+1)=\int_{0}^{m}u^{n_{mix}}(1-u{)}^{n-n_{mix}}du$ is the incomplete beta function. Fig. 2 shows the posterior distribution of *ρ* for a range of values of *E_min_*.

It is important to note that by the Law of Large Numbers, in the limit *n*, *n_mix_*→∞, (1/*n*)*S_D_*→*mρ*. Because we observe *S_D_* = *n_mix_* mixed infection, this implies that *mρ* ≈ *n_mix_*/*n*, thus

(12) $$\rho \to \frac{1}{m}\frac{n_{mix}}{n}\;\text{as}\;n,n_{mix}\to \infty .$$

Alternatively, taking the expectation of the posterior distribution in Eq. (11) and noting that the variance vanishes in the limit yield the same result. Eq. (12) provides a simple estimate for the real prevalence of mixed infection and it suggests that *m* gives a numerical value of the bias coefficient. Figs. 4 and 5 show the details of its behaviour and sensitivity analysis.

### 2.6. Deterministic approximation

If the number of initial bacteria is large for both minority and majority strains (for instance if the initial sample is large), the model can be simplified, removing most of its stochasticity. In this case we consider continuous approximations of the variables *X*_0_ and *Y*_0_:

(13) $$Y_{0}=N(\mu_{Y},\sigma_{Y}^{2}),$$

(14) $$X_{0}=(1-\rho )X_{\left[0,1\right)}+\rho N(E_{min},E_{min})X_{\left[1,+\infty \right)}.$$

Eqs. (13) and (14) can be considered as limit distributions of (8) and (9) respectively because the Gamma and Poisson distributions converge to a normal when the mean is large. In the deterministic approximation, sub-division and growth are not stochastic, yielding

(15) $$X_{T}=\frac{2^{\lambda_{X}T}}{d}X_{0},$$

(16) $$Y_{T}=\frac{2^{\lambda_{Y}T}}{d}Y_{0}.$$

Therefore the slope *m* can be expressed explicitly with the following expression:

$$m=P(X_{T}>fY_{T})=P\left(\frac{2^{\lambda_{X}t}}{d}X_{0}-f\frac{2^{\lambda_{Y}t}}{d}Y_{0}>0\right)\;\;\times \text{evaluated substituting}\;\rho =1\;\text{in Eq}.(14)$$

The substitution *ρ* = 1 follows from the fact that $P(X_{T}>fY_{T})$ is linear with respect to *ρ*, as in Eq. (10). The expression inside the brackets is a linear combination of two normal random variables and therefore is a new normal with known cumulative density function. Therefore

(17) $$m=\frac{1}{2}\left(1-\text{erf}\left(\frac{f2^{(\lambda_{Y}-\lambda_{X})t}\mu_{Y}-E_{min}}{\sqrt{2E_{min}+f^{2}2^{2(\lambda_{Y}-\lambda_{X})t+1}\sigma_{Y}^{2}}}\right)\right).$$

This quantifies the bias in measurements of the prevalence of mixed infection, and how that bias depends on the relative growth rates of minority and majority type cells in culture.

*Parameters*: The parameters and random variables are given in Tables 1 and 2 respectively.

## 3. Results

We computed and analysed the posterior distribution of the prevalence of mixed infection assuming that we observe *n_mix_*/*n* = 15% mixed infection in a study of *n*=500 patients. This baseline estimate of 15% represents a value of mixed infection that is in the range observed in other studies in high TB incidence areas in sub-Saharan Africa (Cohen et al., 2012; Hanekom et al., 2013). Fig. 2 displays a number of different posterior distributions of *ρ* related to the average number of minority type cells per sputum sample *E_min_*. In particular, the smaller the *E_min_* is, the larger the expectation of *ρ* is. This is because numerous opportunities for false negatives arise when the initial population of the minority strain is small or when its growth rate is relatively low. Our posterior estimate accounts for these possible sources of bias, and therefore the estimated mixed infection prevalence may be much higher than the observed 15%. When hosts with mixed infection consistently have a good representation of minority types in their sputum, there are fewer false negatives, *m* is higher, and the posterior estimate of *ρ* is closer to the fraction of cases in whom we detect mixed infection (*n_mix_*/*n*).

**Fig. 3.**
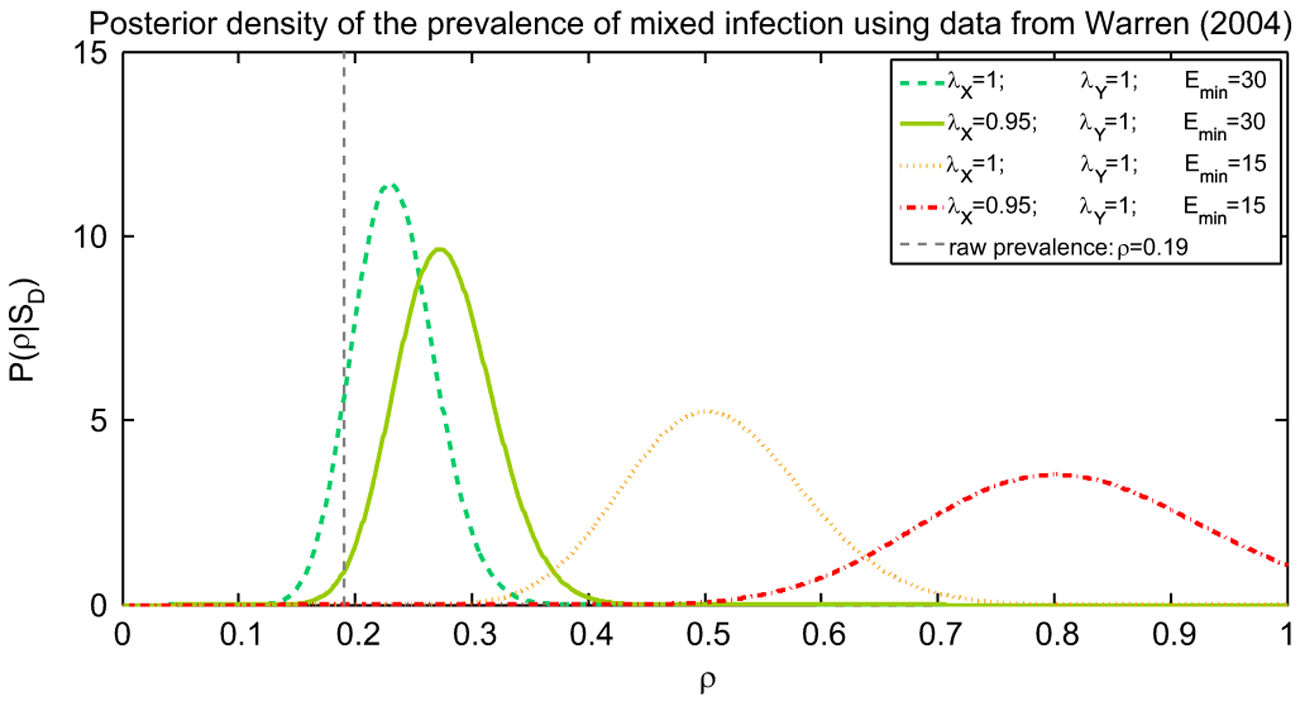
Posterior density distributions $P(\rho |S_{D})$ of the prevalence of mixed infection *ρ*, for different values of the expected number of minority cells in sputum *E_min_*, one optimistic and one pessimistic, and two different combinations of the growth rates of minority and majority type cells, *λ_X_* and *λ_Y_*. We considered *n* = 186 patients, *n_mix_* = 35 of whom are detected with mixed infection, as in Warren et al. (2004). The values for the growth rates are in line with the estimations in Sarkar et al. (2012). The raw estimate from the data would indicate a mixed infection prevalence of approximately *n_mix_*/*n* = 19%, corresponding to *ρ* = 0.19, however we observe that, even in the most optimistic scenario (green dashed line) the posterior *ρ* is higher that is 0.19 with probability 0.9 and has mean over 0.23. (For interpretation of the references to colour in this figure caption, the reader is referred to the web version of this paper.)

We evaluated the posterior distribution of the prevalence of mixed infection in a specific study (Warren et al., 2004) and in Fig. 3 we presented four possible posteriors for optimistic and pessimistic values of *E_min_* and the growth rates. In the most optimistic scenario the posterior *ρ* is higher that 0.19 with probability 0.9 and has mean over 0.23. On the other hand a more moderate choice of parameters would indicate that 0.35 < *ρ* < 0.65 with probability 0.95.

**Fig. 4.**
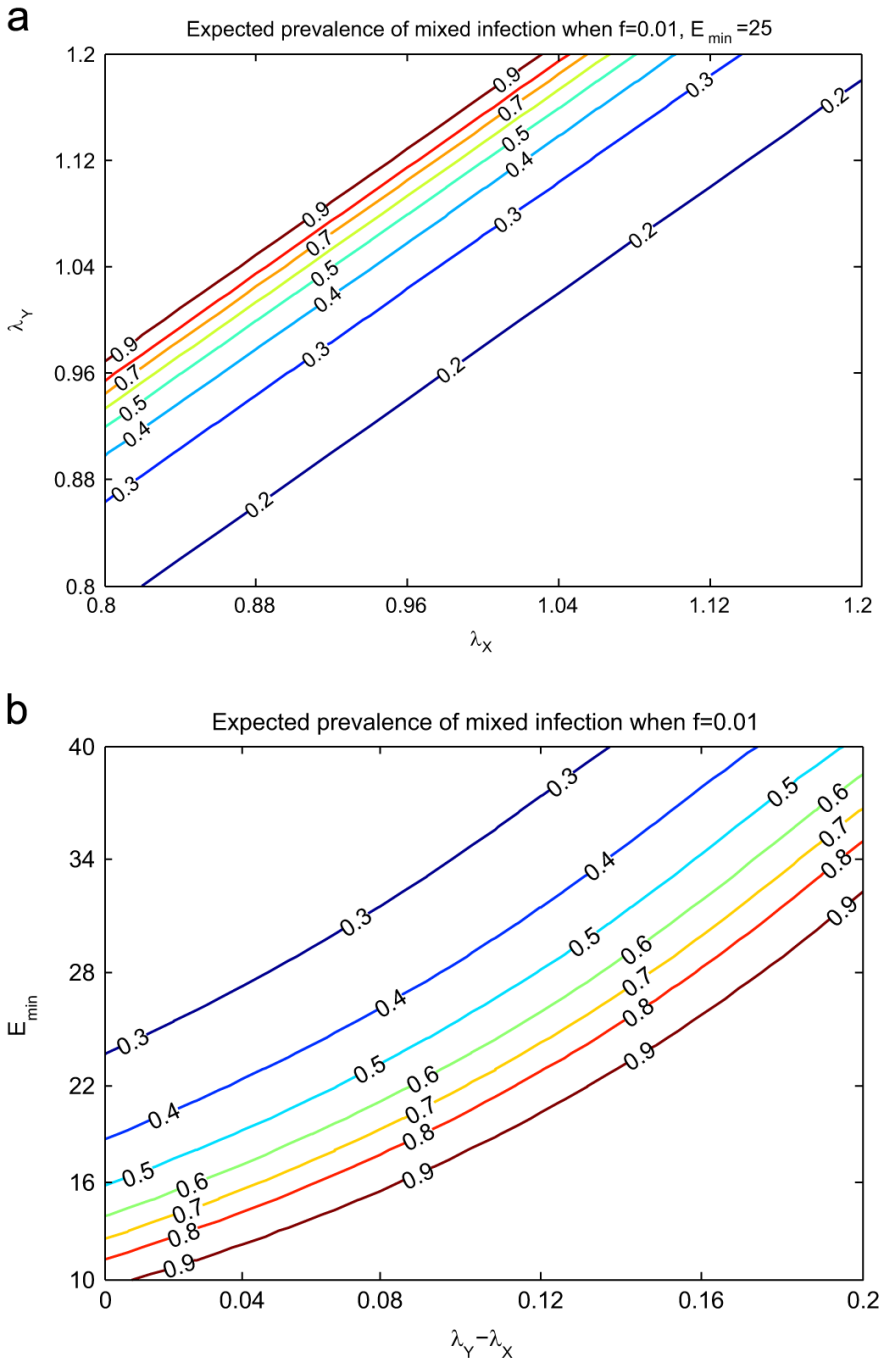
We calculated *E*[*ρ*], the expected prevalence of mixed infection, in a study with *n_mix_* = 75 individuals detected with mixed infection among *n* = 500 patients. In (a) for fixed values of the average number of minority type cells in sputum, *E_min_* = 25, and of the sensitivity threshold *f* = 0.01 we can see a numerical evidence that *E*[*ρ*] depends on the difference of the growth rates *λ_Y_* – *λ_X_* and not on the two growth rates independently; this is confirmed by the deterministic approximation, Eq. (17). In panel (b) how *E*[*ρ*] varies taking into account the difference *λ_Y_* – *λ_X_* on the *x*-axis and the parameter *E_min_* on the *y*-axis is shown. From panel (b) we note that there is a large area (bottom-right) in the parameter space where *E*[*ρ*] is close to 1, estimate very far from the detected 0.15. Although *E*[*ρ*] decrease rapidly from 0.9 to 0.6, most part of the parameter space features an expected prevalence of mixed infection larger than 0.3.

Fig. 4a shows how the estimate of the prevalence of mixed infection *E*[*ρ*] varies for different values of the growth rates. From Fig. 4a we conclude that *E*[*ρ*] does not depend directly on *λ_X_* and *λ_Y_* but on their difference *λ_Y_* – *λ_X_*. This is confirmed by the deterministic approximation and in particular by the expression in Eq. (17) for *m*.

Fig. 4b illustrates the estimate of the prevalence of mixed infection *E*[*ρ*] using a contour plot in the plane (*λ_Y_* – *λ_X_*, *E_min_*). Fig. 4b demonstrates that the bias in detection of mixed infection is related to the number of minority-type bacilli the sputum sample and the difference of the growth rates. It is noteworthy that there is a region of rapid change in the estimate - for example in Fig. 4(b), if *E_min_* is near 20, the estimate is very sensitive to *λ_Y_* – *λ_X_* when the latter is near 0.1. This implies that, in some studies, the raw estimate *n_mix_*/*n* may be uninformative. Independent estimates of *E_min_* and *λ_Y_* – *λ_X_* would greatly improve our ability to interpret such studies.

**Fig. 5.**
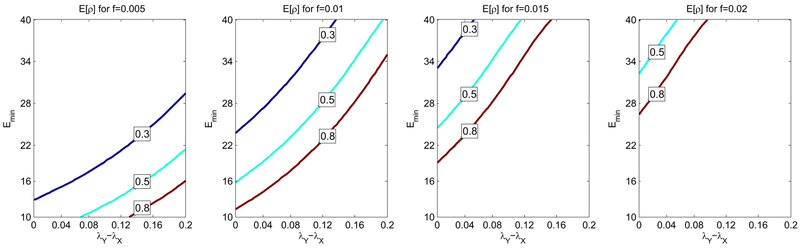
Contour lines of the expected prevalence of mixed infection *E*[*ρ*] are drawn for four different values of the sensitivity threshold *f* of the genotyping method. Every plot shows three elevation levels (0.3, 0.5 and 0.8) when the difference of the growth rates *λ_y_* – *λ_X_* spans between 0 and 0.2 (*x*-axis) and the expected number of minority strain cells in sputum *E_min_* spans between 10 and 40 (*y*-axis). To produce each plot we simulated a study involving 500 patients among whom 75 are detected with mixed infection. From the comparison of the contour plot we evince that as the threshold *f* increases, a greater portion of the parameter space features a high (> 0.8) expected prevalence of mixed infection. On the other hand, when *f* is small, *E*[*ρ*] is closer to the detected prevalence 15%. This not only confirms that a small sensitivity threshold allows more precise results, but also shows that even when such threshold is small, the raw percentage 15% should be corrected to give a good estimate of the real prevalence of mixed infection.

In Fig. 5 the four contour plots of the posterior estimate of mixed infection *E*[*ρ*] for four different values of the sensitivity threshold *f* are compared. In each plot the percentage of detected mixed infection is *n_mix_*/*n* = 19% as in Warren et al. (2004). We can see that as *f* increases, there is a larger area where *E*[*ρ*] > 0.8. This confirms that the higher the sensitivity thresholds is, the higher the chances are of non-detecting mixed infection. Consequently if a percentage *n_mix_*/*n* is detected then it is likely that the real prevalence *ρ* is much higher, even close to 1. It is important to note that even if the sensitivity threshold is reasonably small, see the plot where *f*=0.01, the raw percentage *n_mix_*/*n* is still not a good estimate for a large portion of the parameter set. We conclude that the correction factor 1/*m* is necessary both when *f* is small and when *f* is large.

## 4. Discussion

We developed a mathematical framework both for assessing the conditions under which current methods underestimate the prevalence of mixed infections and for quantifying the potential magnitude of this bias. We found that the prevalence of mixed infection is biased by a factor *m* which depends on the growth rates and the population of the minority strain in the initial samples. With the parameters we have used, for example, if initial mixed infection sputum samples had on average 80 minority type cells per mL, the posterior estimate of the prevalence of mixed infection is 33%, compared to the direct measurement of only 15%.

Our framework combines a binomial model for the specimen sub-division with a birth model for bacterial growth in culture, treating the populations of the minority and majority strains separately. Assuming that detection occurs if and only if the ratio between the two populations is greater than a threshold *f*, we merged the two distributions using the law of total probability. This allowed us to obtain a posterior estimate of the prevalence of mixed infection, represented by the parameter *ρ*. We found that stochastic effects during specimen handling may reduce the probability of detecting mixed infections. On the other hand if the sample size were increased, fewer stochastic effects would interfere with the detection of mixed infection and the raw percentage could be a more accurate estimate.

The parameter *m*, and therefore the distribution of *ρ*, is very sensitive to variation of *λ_Y_* and *λ_X_*. The growth rates and, more importantly, their difference are usually not known and have important consequences for our ability to observe mixed infections in culture. Targeted experiments to measure the growth rates could help inform the extent of bias in estimation of mixed infection. These experiments could be done if it were possible to resample from initial cultures to obtain cells of both types to measure absolute and relative growth rates in culture. The parameter *E_min_*, the expected number of minority cells in the specimen given that the host has mixed infection, also affects the distribution of *ρ* and therefore the bias, as shown in Fig. 2. In this paper we decided to treat *E_min_* as a parameter and not as another random variable. In fact we have not modelled the specimen collection, but only the specimen handling: *E_min_* has to be interpreted as reflecting the numbers of minority strain bacilli which, if present, will arise in the sputum sample, and this is beyond the scope of this paper. However, the diversity of TB present in a host is potentially complex and heterogeneously distributed, comprising some clonal diversity (Colijn et al., 2011) in addition to diversity resulting from multiple infections. It is reasonable to suspect that not all of the diversity will be represented in sputum samples, and that this is an additional source of bias in detecting mixed infections.

The model presented in this paper is limited in its complexity. Here, we only consider a minority and a majority strain while in reality there may be more than two different strains. Moreover we consider only strains that potentially can be detected with genotyping, i.e. strains with different MIRU types. In a real situation there can be a reinfection with bacteria having the same MIRU type and, therefore, it is impossible to detect such mixed infections with genotyping. New studies which use methods with additional sensitivity for detecting variation between strains, such as whole genome sequencing, will likely be increasingly used to understand within-host diversity (Sun et al., 2012; Chan et al., 2013; Köser et al., 2013). However, it is important to recognize that most studies will continue to be limited by the examination of sputum samples, which may not represent the actual degree of strain heterogeneity within a host (Cohen et al., 2011). These examples suggest that mixed infections can be even more frequent than in the results reported here. On the other hand, our results also suggest that when the population size of the minority strain is large, > 3%, bias is minimal and the detected prevalence of mixed infection is very close to the real prevalence.

Mixed infection is of interest because it is informative of aspects of the epidemiology of tuberculosis, but it may be particularly relevant to the estimation of the prevalence and infectiousness of drug-resistant TB strains. Drug-sensitive and drug-resistant strains of TB can compete for susceptible hosts, and can re-infect hosts who already have one strain of TB, resulting in mixed infections. A higher estimated incidence of mixed infection could therefore suggest new estimates of the extent of reinfection, and of the level of transmission of resistant strains.

Mixed infections have been detected in nearly 15% of cases in a number of studies (Cohen et al., 2012; Hanekom et al., 2013), and have been considered to play an important role in facilitating the stable coexistence of different strains (Colijn et al., 2009), in altering treatment outcomes (van Rie et al., 2005) and undermining the effectiveness of TB control programmes (Cohen et al., 2008). In this paper we provide strong evidence that estimates of the prevalence of mixed infection can be considerably higher than the raw detection frequency. This implies that mixed infection could play an even more important role in TB epidemiology than raw estimates would suggest.

## Supplementary Material

1

## Figures and Tables

**Table 1:** Parameters.

Parameters	Description	Range/expression
*ρ*	Probability of presence of mixed infection in sputum sample	Eq. (9)
*E_min_*	Mean of minority type cell in the sputum sample given presence of mixed infection	1–40
*λ_X_*	Growth rate of minority strain cells	1–1.2, from Sarkar et al. (2012)
*λ_Y_*	Growth rate of majority strain cells	1–1.2, from Sarkar et al. (2012)
*T*	Growth time	7 days
**l**	Exponent of the approximation function (3)	0.336 (see Supplement)
*C*	Coefficient in the approximation function (3)	Supplement Eqs. (8) and (10)
*f*	Threshold for detection of mixed infection	0.005–0.02
*d*	Number of parts the sputum sample is divided in during handling	4

**Table 2:** Random variables.

Random variable	Description	Expression
X_0_	Number of minority strain cells in the sputum sample assumed mean: *ρE_mix_*	Eq. (9)
*Y*_0_	Number of majority strain cells in the sputum sample assumed mean: 1750 from Core Curriculum for Disease Control (), Palaci et al. (2007)	Eq. (8)
*X_T_*	Number of minority strain cells after specimen handling	Eq. (2)
*Y_T_*	Number of majority strain cells after specimen handling	Eq. (4)
*D*	Bernoulli random variable representing the test result for mixed infection	Eq. (6)
*S_D_*	Total mixed infection detected in a study with *h* patients	Eq. (7)

## AUTHOR-HIGHLIGHTS

- We develop a mathematical model for study designs aimed to detect TB mixed infection.
- We obtain Bayesian posterior estimates of the prevalence of mixed infection.
- The bias between the posterior estimate and the observed prevalence is discussed.
- The posterior estimate can be substantially higher than the raw percentage.
