# Supplementary material for: Magnitude and sources of bias in the detection of mixed strain M. tuberculosis infection
Source: J Theor Biol. Author manuscript; Available in PMC 2020 Feb 11. (PMC7011203; doi:10.1016/j.jtbi.2014.12.009)
Supplement: 1 [file NIHMS1516474-supplement-1.pdf]

Supplement

Derivation of the distribution of X_T

A sputum sample includes X_0 minority strain bacteria who undergo sub-division phase. After sub-division a colony originates from each bacterium left. To simplify the notation we introduce the random variables Col_X, representing the number of colonies, and $Size_j$, representing the size of the j-th colony at time T. It follows:

$$X_T = \sum_{j=1}^{Col_X} Size_j.$$

The sub-division phase is a binomial choice, so:

$$\mathbb{P}(Col_X = i) = \binom{X_0}{i}\left(\frac{1}{d}\right)^i \left(\frac{d-1}{d}\right)^{X_0-i}, \; i = 0, ..., X_0 \tag{1}$$

Each bacterium starts a colony whose size is modeled by a birth-only process. X_T is therefore modeled by a birth-only process which starts with Col_X cells. Birth-only processes follow a negative binomial distribution, it follows:

$$\mathbb{P}(X_T = k | Col_X = i) = \binom{k-1}{i-1} p_X^i (1-p_X)^{k-i} \tag{2}$$

where $p_X = 2^{-\lambda_X T}$. Using law of total probability:

$$\mathbb{P}(X_T = k) = \sum_{i=1}^{\min(X_0,k)} \mathbb{P}(M = k | Col_M = i)\mathbb{P}(Col_M = i). \tag{3}$$

The apex of the sum derives from the fact that there cannot be less bacteria than the number of colonies, as we do not consider death. In fact $\mathbb{P}(X_T = k | Col_X = i) > 0$ with $k < i$ only if some colonies die. Substituting Equations (1) and (2) in (3) we obtain:

$$\mathbb{P}(X_T = k) = \sum_{i=1}^{\min(X_0,k)} \binom{k-1}{i-1}\binom{X_0}{i} p_X^i (1-p_X)^{k-i} \left(\frac{1}{d}\right)^i \left(\frac{d-1}{d}\right)^{X_0-i} \tag{4}$$

The approximation of the distribution of X_T

Using Stirling's formula, it follows that as $k \to \infty$:

$$(k-1)! \approx \sqrt{2\pi(k-1)}\left(\frac{k-1}{\mathrm{e}}\right)^{k-1},$$

$$(k-i)! \approx \sqrt{2\pi(k-i)}\left(\frac{k-i}{\mathrm{e}}\right)^{k-i}.$$

The second relation above is valid because $i \leq X_0 \ll k$, from equation (4) in the paper. Because k is large:

$$(k-1)^{k-1} = k^{k-1}\left(1-\frac{1}{k}\right)^k\left(1-\frac{1}{k}\right)^{-1} \approx k^{k-1}\mathrm{e}^{-1}\left(1-\frac{1}{2k}\right)\left(1-\frac{1}{k}\right)^{-1} \approx \frac{k^k}{\mathrm{e}(k-1)}\frac{2k-1}{2k}$$

And in a similar way:

$$(k-i)^{k-i} \approx \frac{k^k}{\mathrm{e}^i (k-i)^i}\frac{2k-i^2}{2k}$$

Therefore:

$$\binom{k-1}{i-1} = \frac{(k-1)!}{(k-i)!(i-1)!} \approx \frac{1}{(i-1)!}\sqrt{\frac{k-1}{k-i}}\frac{(k-i)^i}{k-1}\left(1+\frac{i^2-1}{2k-i^2}\right). \tag{5}$$

Substituting (5) in the distribution of X_T (equation (4) in the paper) yields:

$$\begin{aligned}
\mathbb{P}(X_T = k) &\approx \sum_{i=1}^{X_0} \frac{1}{(i-1)!}\sqrt{\frac{k-1}{k-i}}\frac{(k-i)^i}{k-1}\left(1+\frac{i^2-1}{2k-i^2}\right)\binom{X_0}{i}\frac{p_X^i}{(1-p_X)^{i-k}}\left(\frac{1}{d}\right)^i\left(\frac{d-1}{d}\right)^{X_0-i} \\
&\approx \sum_{i=1}^{X_0} \frac{(1-p_X)^k}{\sqrt{k-1}}\frac{(k-i)^i}{\sqrt{k-i}}\frac{p_X^i}{(1-p_X)^i}\frac{1}{(i-1)!}\left(1+\frac{i^2-1}{2k-i^2}\right)\binom{X_0}{i}\left(\frac{1}{d}\right)^i\left(\frac{d-1}{d}\right)^{X_0-i} \\
&\approx \frac{(1-p_X)^k}{\sqrt{k-1}}\sum_{i=1}^{X_0}\frac{(k-i)^i}{\sqrt{k-i}}\left(1+\frac{i^2-1}{2k-i^2}\right)\boxed{\frac{p_X^i}{(1-p_X)^i}\frac{1}{(i-1)!}\binom{X_0}{i}\left(\frac{1}{d}\right)^i\left(\frac{d-1}{d}\right)^{X_0-i}} \\
&\approx \frac{(1-p_X)^k}{\sqrt{k-1}}\sum_{i=1}^{X_0}\frac{(k-i)^i}{\sqrt{k-i}}\left(1+\frac{i^2-1}{2k-i^2}\right)H_i
\end{aligned}$$

where H_i indicates the boxed factor. Note that:

$$\left(1+\frac{i^2-1}{2k-i^2}\right) \approx 1,$$

$$\frac{\partial (k-i)^{i-1/2}}{\partial i} = (k-i)^{i-1/2}\left[\log(k-i) - \frac{i-1/2}{k-i}\right].$$

Because $k \gg X_0$, also $k \gg i$ because $i \leq X_0$. If $k \gg i$ we can consider the above derivative positive and therefore the term $(k-i)^{i-1/2}$ is increasing as i increases. Because i varies from 1 to X_0 we can consider the following bounds:

$$(k-1)^{1/2} \leq (k-i)^{i-1/2} \leq (k-X_0)^{X_0-1/2} < (k-1)^{X_0-1/2}$$

We can now obtain bounds for the probabilities, in the limit $k \gg X_0$:

$$\begin{aligned}
\mathbb{P}(X_T = k) &\leq \frac{(1-p_X)^k}{(k-1)^{1/2}}\sum_{i=1}^{X_0}(k-1)^{X_0-1/2}H_i \\
&= (1-p_X)^k(k-1)^{X_0-1}\sum_{i=1}^{X_0}H_i \\
&\leq C(1-p_X)^k(k-1)^{X_0-1}
\end{aligned} \tag{6}$$

$$\mathbb{P}(X_T = k) \geq \frac{(1-p_X)^k}{\cancel{(k-1)^{1/2}}}\sum_{i=1}^{X_0}\cancel{(k-1)^{1/2}}H_i = C(1-p_X)^k, \tag{7}$$

where

$$C = \sum_{i=1}^{X_0} H_i. \tag{8}$$

Observing the bounds (6) and (7) we seek an approximation for the distribution of X_T of the form:

$$\mathbb{P}(X_T = k) \approx C(1-p_X)^k (k-1)^{l(X_0-1)}, \text{ with } 0 \le l \le 1. \tag{9}$$

We compared graphically the behaviour of the approximation (9) and the frequency of the simulation, and we found that when k is bigger than the third quartile q_3 the approximation (9) is valid. Therefore we can set:

$$\sum_{k=q_3}^{\infty} C(1-p_X)^k (k-1)^{l(X_0-1)} = \frac{1}{4}$$

$$\sum_{i=0}^{\infty}(1-p_X)^i (i-1)^{l(X_0-1)} - \sum_{i=0}^{q_3-1}(1-p_X)^i (i-1)^{l(X_0-1)} = \frac{1}{4}C^{-1}$$

$$C = \frac{1}{4}\left[\Phi\big(1-p_X, -1, l(1-X_0)\big) - \sum_{i=0}^{q_3-1}(1-p_X)^i (i-1)^{l(X_0-1)}\right]^{-1}, \tag{10}$$

where Φ is the Lerch Transcendent Zeta Function [15], defined by the above series. In this way we expressed the constant C independently from the challenging computation of the sum of the H_i-s. Substituting (10) in (9) we obtain a class of functions parametrized by l. We define the parametric model:

$$\{f_{l^*}(k) = C(l^*)(1-p_X)^k (k-1)^{l^*(X_0-1)}, l^* \in (0,1)\}.$$

Using Maximum Likelihood Estimation (MLE) we want to find the value $l \in (0,1)$ such that $\mathbb{P}(X_T = k) \approx f_l(k)$ for $k \gg X_0$. MLE is usually employed to seek an entire distribution and not only an asymptotic behavior. First we run number of simulations of X_T with `Matlab` in order to obtain a sample (of X_T): $(m_1, ..., m_h)$. As we discussed before, our approximation is valid for $k > q_3$, therefore we restrict MLE to the m_i-s that are bigger than the sample 75th percentile q_3. Using q_3 we can compute the parameter C from (10) and with the sample results we can compute the approximation (9). Now we can use the Maximum Likelihood Principle, i.e. we need to solve the optimization problem:

$$l = \left\{ \max \left(\prod_{m_i \ge q_3} f_{l^*}(m_i) \right) \text{ with } l^* \in (0,1) \right\}.$$

The maximum l, if it exists, is then the value that substituted in (9) gives the best approximation. With `Matlab`, function `max`, we find the value l maximizing the logarithm of the product, to improve speed and accuracy (the product can be very large). Moreover we found that the maximum l converges as the simulation size increases:

$$\begin{aligned} &l \to 0.608 \text{ if the sample is divided in } d = 2 \text{ parts} \\ &l \to 0.336 \text{ if the sample is divided in } d = 4 \text{ parts} \\ &l \to 0.206 \text{ if the sample is divided in } d = 6 \text{ parts} \end{aligned} \tag{11}$$

Using l as in (11) and C as in (10), we can compute the approximation of the probability distribution (9). Figure S1 shows the behaviour of l and C varying X_0, T and λ_X: the parameter l can be taken as constant.

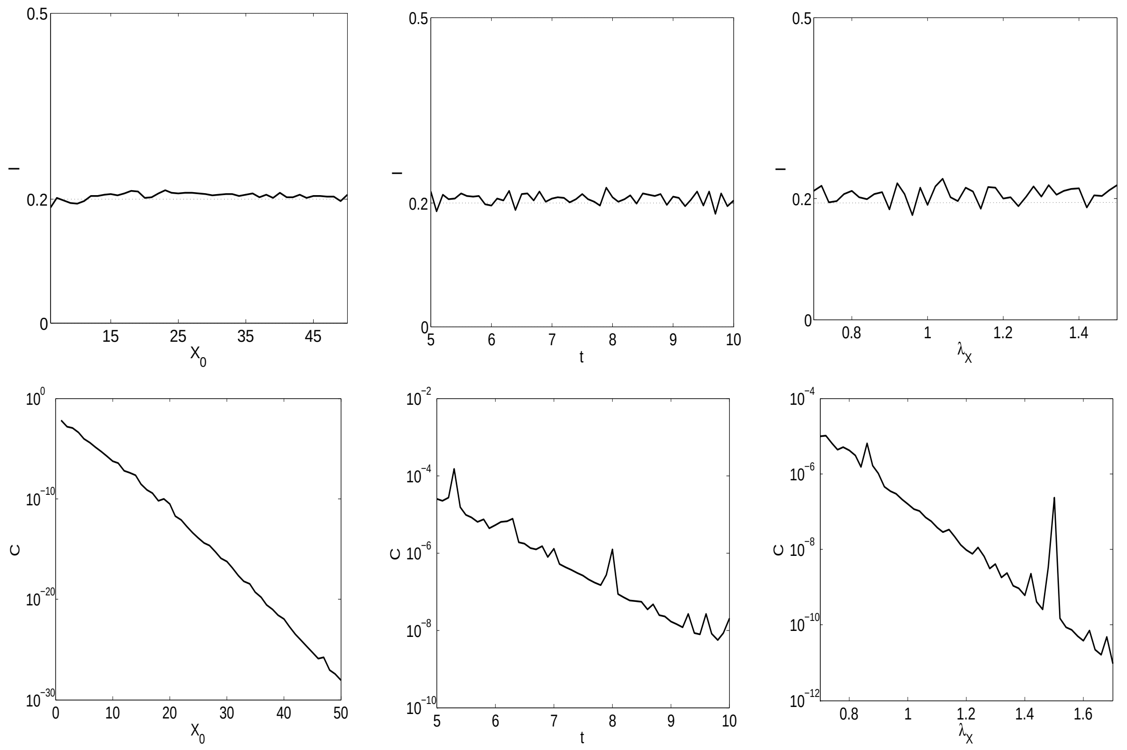

Figure S1: Behaviour of the parameters l and C as functions of the initial number of minority type cells X_0, the growth time T and the growth rate λ_X; in each plot we assume that the initial sputum sample is divided in $d = 6$ parts. The parameter l is calculated using maximum likelihood estimation; the parameter C is evaluated from equation (10). The graphs show that l remains constant, whereas C decrease exponentially.

Our approximation function is therefore:

$$App(k|X_0) = \begin{cases} \mathbb{P}(X_T = k|X_0) & \text{if } k \leq q_3 \\ C(1-p_X)^k (k-1)^{l(X_0-1)} & \text{if } k \geq q_3 \end{cases} \tag{12}$$

Figure S2a compares the approximation with the simulation of the distribution of X_T.

The approximation of the distribution of Y_T

We recall that the analytic expression of $\mathbb{P}(Y_T = k)$ is:

$$\mathbb{P}(Y_T = k) = \sum_{i=1}^{\min(Y_0,k)} \binom{k-1}{i-1} \binom{Y_0}{i} p_Y^i (1-p_Y)^{k-i} \left(\frac{1}{d}\right)^i \left(\frac{d-1}{d}\right)^{Y_0-i} \tag{13}$$

We want to find an approximation of (13) in the case $1 \ll Y_0 < \infty$. Recall also:

$$\mathbb{P}(Y_T = k) = \sum_{i=1}^{\min(Y_0,k)} \mathbb{P}(Y_T = k|Col_Y = i)\mathbb{P}(Col_Y = i), \tag{14}$$

where $\mathbb{P}(Y_T = k|Col_Y)$ is a negative binomial and $\mathbb{P}(Col_Y = i)$ is a binomial distribution representing the number of colonies. We have:

$$Y_T := \sum_{i=1}^{Col_Y} Size_i. \tag{15}$$

The above can be written in the following form:

$$Y_T = \left(\frac{1}{Col_Y} \sum_{i=1}^{Col_Y} Size_i \right) Col_Y. \tag{16}$$

In order to simplify the notation we introduce: $A := \left(\frac{1}{Col_Y} \sum_{i=1}^{Col_Y} Size_i \right)$. We will now prove that $A \to E[Size]$ in probability as $Y_0 \to \infty$. Firstly we note that since Col_Y is a binomial random variable), for any $c < \infty$ it verifies the following:

$$\lim_{Y_0 \to \infty} \mathbb{P}\left(Col_Y < c\right) = 0. \tag{17}$$

This means:

$$\forall \delta_1 > 0 \ \exists M \ \text{ s.t. } \forall Y_0 > M : \mathbb{P}\left(Col_Y < c\right) < \delta_1. \tag{18}$$

Now we recall the Weak Law of Large Numbers and we apply it to $Size$. For any $\epsilon > 0$, WLLN states that:

$$\lim_{r \to \infty} \mathbb{P}\left(\left| \frac{1}{r} \sum_{i=1}^{r} Size_i - E[Size] \right| > \epsilon \right) = 0, \tag{19}$$

which means:

$$\forall \delta_2 > 0 \ \exists \ \overline{r} \ \text{ s.t. } \forall r > \overline{r} : \mathbb{P}\left(\left| \frac{1}{r} \sum_{i=1}^{r} Size_i - E[Size] \right| > \epsilon \right) < \delta_2. \tag{20}$$

Now we choose c of equation (18) equal to $\overline{r}$ of equation (20). Therefore for $Y_0 > M$: $\mathbb{P}(Col_Y < \overline{r}) < \delta_1$. Moreover, from the Law of Total Probability

$$\begin{aligned} \mathbb{P}(|A - E[Size]|) =& \mathbb{P}(|A - E[Size]| \big| Col < \overline{r})\mathbb{P}(Col < \overline{r}) + \\ &+ \mathbb{P}(|A - E[Size]| \big| Col \geq \overline{r})\mathbb{P}(Col \geq \overline{r}). \end{aligned} \tag{21}$$

Therefore, combining (18) and (20) and using (21):

$$\forall \delta_1, \delta_2 > 0 \ \exists \ M \ \text{ s.t. } \forall Y_0 > M : \mathbb{P}(|A - E[Size]| > \epsilon) < \delta_1 + \delta_2. \tag{22}$$

Since δ_1 and δ_2 are arbitrary, equation (22) implies that $A \to E[Size]$ in probability.

Using known results in probability theory, the binomial distribution Col can be approximated by a normal distribution as $Y_0 \to \infty$. Since the factor A converges to $E[Size]$ in probability, thus in distribution, we conclude that the distribution of Y_T can be approximated with a normal.

Now we need to find the exact mean and variance of N. We already know that:

$$E[Size] = 2^{\lambda_Y T} \ , \ var(Size) = 2^{\lambda_Y T}(2^{\lambda_Y T} - 1) \ , \ E[Col] = \frac{Y_0}{d} \ , \ var(Col) = \frac{d(d-1)}{d^2} Y_0.$$

Since $Size$ and Col are independent, the expectation of Y is found to be:

$$E[Y_T] = \frac{Y_0}{6} 2^{\lambda_Y T}. \tag{23}$$

To find the variance, we will make use of the Law of Total Variance:

$$\begin{aligned} var(Y_T) &= E[var(Y_T|Col)] + var(E[Y_T|Col]) \\ &= E[Col \cdot var(Size)] + var(Col \cdot E[Y_T]) \\ &= E[Col]var(Y_T) + \big(E[Y_T]\big)^2 var(Col) \\ &= \frac{Y_0}{d} 2^{\lambda_Y T}(2^{\lambda_Y T} - 1) + \frac{Y_0 d(d-1)}{d^2} 4^{\lambda_Y T}. \end{aligned} \tag{24}$$

Figure S2b compares the normal approximation with the simulation of the probability of Y_T.

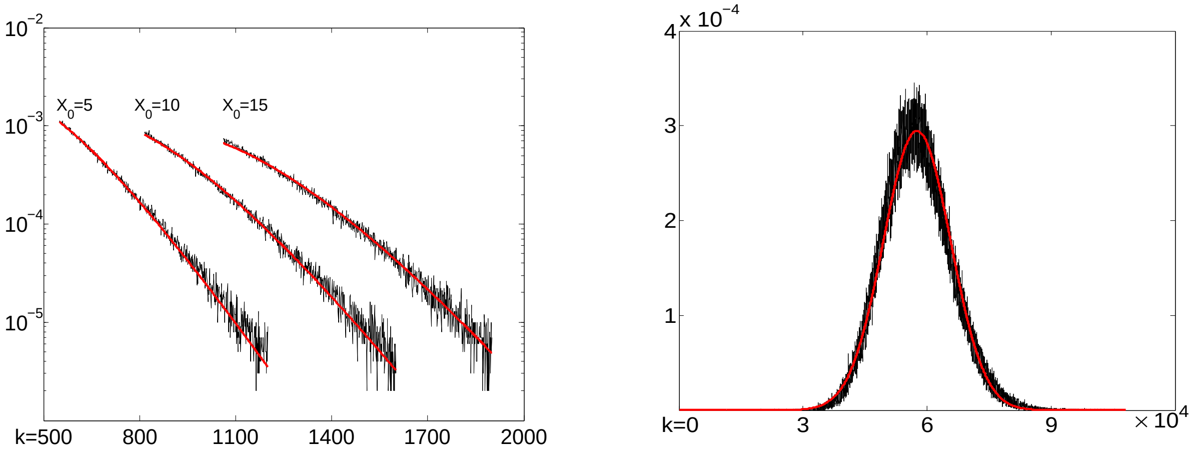

(a) Comparison between the approximation (equation (9), red line) and the simulation of the distribution of X_T (equation (4), black line).

(b) Comparison between the normal approximation (red line) with mean and variance from equation (23) and (24) and the simulation of the distribution of Y_T (equation (13), black line).

Figure S2: The comparisons show that the approximations are close to the real distributions

Connecting X_T and Y_T to detection

To find the distribution of Y_T with no dependence on Y_0 we use LTP. Since numerically: $\mathbb{P}(Y_0 \leq 2500) \approx 1$, we can limit the sum, which otherwise would become computationally intensive:

$$\mathbb{P}(Y_T = k) \approx \sum_{x=0}^{2500} \mathcal{N}(k)\mathbb{P}(Y_0 = x), \tag{25}$$

where $\mathcal{N}$ is the normal approximation. The same principle holds when dealing with the distribution of X_T, but this will still depend on ρ. Since $\mathbb{P}(X_0 \leq 50) \approx 1$, the approximated law of total probability becomes:

$$\mathbb{P}(X_T = k|\rho) \approx \sum_{x=0}^{50} App(k|X_0 = x)\mathbb{P}(X_0 = x|\rho). \tag{26}$$

To find the probability $\mathbb{P}(D = 0) = \mathbb{P}(X_T/Y_T < f)$ we use again LTP:

$$\mathbb{P}(D = 0) = \sum_{k=0}^{\infty} \mathbb{P}(X_T < kf)\mathbb{P}(Y_T = k). \tag{27}$$

In place of $\mathbb{P}(Y_T = k)$ and $\mathbb{P}(X_T < kf)$ we use the approximations found before. In order to limit the sum we note that $\mathbb{P}(Y_T < 120000) \approx 1$ and using the same idea as before:

$$\mathbb{P}(X_T/Y_T < f|\rho) \approx \sum_{k=0}^{1.2\cdot 10^5} \mathbb{P}(X_T < fk|\rho)\mathbb{P}(Y_T = k). \tag{28}$$

Details of the Bayesian analysis

Given that $\mathbb{P}(D = 1) = m\rho$, the probability of detecting n_{mix} mixed infections in a study involving n patients is:

$$\mathbb{P}(S_D = n_{mix}|\rho) = \binom{n_{mix}}{n}(m\rho)^{n_{mix}}(1 - m\rho)^{n-n_{mix}}. \tag{29}$$

Equation (29) is the likelihood. Since the likelihood is a binomial, the conjugate prior is given by the Beta distribution, keeping in mind that we need to define it over $m\rho$ and not only ρ:

$$\mathbb{P}(m\rho) \propto (m\rho)^{a-1}(1 - m\rho)^{b-1}\mathcal{X}_{[0,m]}. \tag{30}$$

To keep the prior as uninformative as possible, a and b must be chosen close to 1 where the Beta distribution tends to a uniform distribution. The posterior for $m\rho$ is the truncated beta distribution:

$$\mathbb{P}(m\rho|S_D) = \frac{(m\rho)^{n_{mix}}(1 - m\rho)^{n-n_{mix}}}{\mathcal{B}_m(n_{mix} + 1, n - n_{mix} + 1)}, \tag{31}$$

where $\mathcal{B}_m(n_{mix} + 1, n - n_{mix} + 1) = \int_0^m u^{n_{mix}}(1 - u)^{n-n_{mix}}du$ is the incomplete beta function. Now note that $\text{pmf}_{m\rho}(mx) = \text{pmf}_{\rho}(x)$ and therefore the posterior distribution of ρ is:

$$\mathbb{P}(\rho|S_D) = \frac{(m\rho)^{n_{mix}}(1 - m\rho)^{n-n_{mix}}}{\mathcal{B}_m(n_{mix} + 1, n - n_{mix} + 1)}, \tag{32}$$

The expectation of the posterior is $E[\rho] = \frac{1}{m}E[m\rho] = \frac{1}{m}\frac{\mathcal{B}_m(A+1,B)}{\mathcal{B}_m(A,B)}$. and using properties of the incomplete beta function:

$$E[m\rho|S_D] = \frac{n_{mix} + 1}{n + 2} - \frac{m^{n_{mix}+1}(1 - m)^{n-n_{mix}+1}}{(n + 2)\mathcal{B}_m(n_{mix} + 1, n - n_{mix} + 1)}. \tag{33}$$

Note that[1] the second addend vanishes as $n \to \infty$, and consequently for large n:

$$E[m\rho|S_D] \to \frac{n_{mix} + 1}{n + 2} \to \frac{n_{mix}}{n} \text{ as } n, n_{mix} \to \infty. \tag{34}$$

It follows directly that

$$E[\rho|S_D] \to \frac{1}{m}\frac{n_{mix}}{n} \text{ as } n, n_{mix} \to \infty. \tag{35}$$

In equation (35), $\frac{1}{m}\frac{n_{mix}}{n}$ does not exceed 1 as n_{mix}/n must be smaller than m because, as $n, n_{mix} \to \infty$, it is the expectation of $m\rho$ and $\rho < 1$.

---

[1]Because n and n_{mix} are integers, the incomplete Beta function can be written as $\mathcal{B}_m(n_{mix} + 1, n - n_{mix} + 1) = \mathcal{B}(n_{mix} + 1, n - n_{mix} + 1) \sum_{j=n_{mix}+1}^{n+1} \binom{n+1}{j} m^j(1 - m)^{n+1-j}$. Then: $\mathcal{B}_m(n_{mix} + 1, n - n_{mix} + 1) > m^{n_{mix}+1}(1 - m)^{n-n_{mix}}$. Therefore $\frac{m^{n_{mix}+1}(1-m)^{n-n_{mix}+1}}{(n+2)\mathcal{B}_m(n_{mix}+1,n-n_{mix}+1)} < \frac{1}{n+2} \to 0$ as $n \to \infty$.

Supplementary Results

Figure S3 shows the dependence of m, the probability that a known mixed infection will be detected, on model parameters. We note that strong dependence on the difference between the growth rates and the dependence on E_{min}. Higher values of m correspond to fewer false negatives; as m approaches 1, the posterior estimate of the prevalence of mixed infection approaches the naive estimate n_{mix}/n (i.e. the fraction of detected cases with mixed infection).

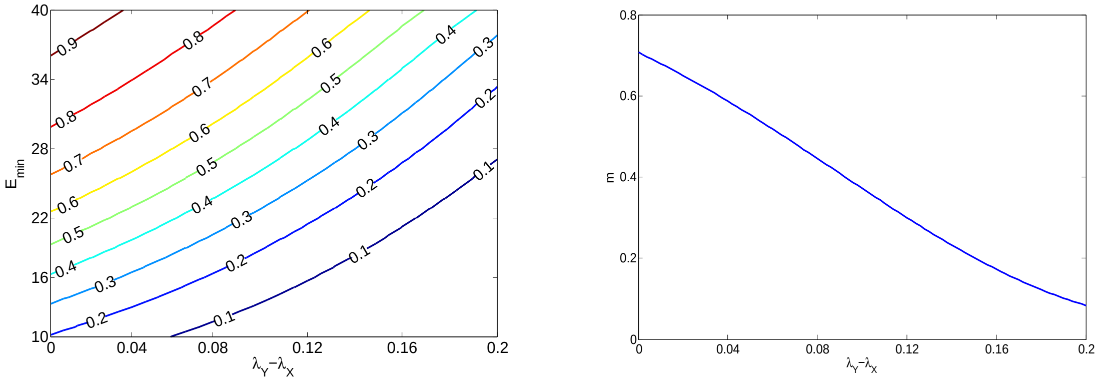

(a) m as a function of $\lambda_Y - \lambda_X$ and E_{min}

(b) m as a function of $\lambda_Y - \lambda_X$, for $E_{min} = 25$

Figure S3: The probability that a known mixed infection is detected (m) as a function of the model parameters. The parameter m is evaluated from its definition, i.e. the probability that, after division and growth, the ratio between minority and majority type cells is larger than the sensibility threshold $f = 0.01$ of the genotyping method. We used a number of divisions $d = 2$.
